# Unprotected Left Main Percutaneous Coronary Intervention: Integrated Use of Fractional Flow Reserve and Intravascular Ultrasound

**DOI:** 10.1161/JAHA.112.004556

**Published:** 2012-12-19

**Authors:** Seung‐Jung Park, Jung‐Min Ahn, Soo‐Jin Kang

**Affiliations:** 1 Heart Institute, Asan Medical Center, College of Medicine, University of Ulsan, Seoul, Korea (S.J.P., J.M.A., S.J.K.)

**Keywords:** fractional flow reserve, intravascular ultrasound, left main stenosis, stent

## Introduction

For several decades, bypass surgery has been regarded as the treatment of choice for patients with unprotected left main coronary artery (LMCA) disease.^1–2^ However, because of easy anatomic accessibility and a relatively large vessel caliber, left main percutaneous coronary intervention (PCI) for LMCA disease has become an attractive option for cardiologists. In addition, technical advances in PCI and stent technology have emboldened physicians to test the feasibility of LMCA intervention and, coupled with the widespread availability of drug‐eluting stents (DESs), has led to reevaluation of the role of PCI as a viable alternative treatment for unprotected LMCA disease.^3^ As a result, during the last decade, the prevalence of LMCA stenting has significantly increased worldwide. In addition, several recent large registries^4–13^ and randomized controlled trials^14–17^ have demonstrated that LMCA stenting yields mortality and morbidity rates comparable to coronary artery bypass grafting (CABG).

Hence, it is timely to now move the discussion forward on how to optimize PCI results, beyond the feasibility and safety issues with LMCA stenting. In this review, we briefly summarize the current status of LMCA stenting and discuss the concept of optimal LMCA stenting through integrated use of fractional flow reserve (FFR) and intravascular ultrasound (IVUS).

## Current Status of LMCA Stenting

On the basis of increasing off‐label experiences with stenting and clinical studies,^4–22^ the American Heart Association/American College of Cardiology^23^ and the European Society of Cardiology and the European Association for Cardio‐Thoracic Surgery.^24^ recently updated the PCI guideline for the elective treatment of LMCA stenosis to include class IIa indications with a B level of evidence depending on the anatomical complexity of the coronary artery disease. Therefore, DES implantation is currently considered an alternative option for selected patients with unprotected LMCA disease. Table 1 summarizes key observational studies, meta‐analyses, and randomized trials that compare PCI with DES and CABG.^4–22^ Detailed guidelines are summarized in Table 2. However, many unresolved technical issues remain, including how to assess the functional significance of intermediate LMCA stenosis and how to optimize procedural outcomes, especially for LMCA bifurcation lesion PCI. In this regard, daily practice has already changed to include more use of FFR and IVUS for LMCA stenting.^25–26^

**Table 1.** Key Comparative Studies of PCI and CABG for Left Main Disease

Design	Contributing Studies	PCI (n)	CABG (n)	Follow‐up Duration	Adjusted Risk for Death	Adjusted Risk for TVR/TLR
Observational study	MAIN‐COMPARE^4^	784	690	5 years	HR 1.00 (0.73 to 0.37) *P*=0.99	HR 6.45 (3.75 to 11.09) *P*=0.34
	Lee et al^5^	153	50	6.7 months	4% for PCI 13% for CABG *P*=0.18	7% for PCI 1% for CABG *P*=0.22
	Chieffo et al^6^	107	142	1 year	OR 0.33 (0.06 to 1.40) *P*=0.17	OR 4.22 (1.49 to 14.55) *P*=0.005
	Palmerini et al^7^	94	154	1.2 years	HR 0.99 (0.47 to 2.07) *P*=0.97	25.5% for PCI 2.6% for CABG *P*=0.0001
	Sanmartin et al^8^	96	245	1 year	5.2% for PCI 8.4% for CABG *P*=0.34	5.2% for PCI 0.8% for CABG *P*=0.004
	Makikallio et al^9^	49	238	1 year	4% for PCI 11% for CABG *P*=0.14	4% for PCI 2% for CABG *P*=0.29
	Cheng et al^10^	94	216	3 years	12.1% for PCI 21.1% for CABG *P*=0.01	16.0% for PCI 6.1% for CABG *P*=0.002
	Wu et al^11^	131	245	3 years	HR 0.22 (0.06 to 0.81) *P*=0.02	HR 2.69 (1.30 to 5.57) *P*=0.008
	Park et al^12^	205	257	3 years	OR 1.20 (0.70 to 2.08) *P*=0.51	OR 5.56 (2.85 to 10) *P*<0.001
	CUSTOMIZE^13^	222	361	1 year	HR 1.1 (0.4 to 3.0) *P*=0.81	HR 8.0 (2.2 to 28.7) *P*=0.001
Meta‐analysis	Takagi et al^19^	1006	1175	3 months to 3 years	OR 0.99 (0.69 to 1.43) *P*=0.97	OR 5.05 (3.07 to 8.30) *P*<0.001
	Lee et al^20^	1236	1669	1 year	OR 0.83 (0.64 to 1.25)	OR 2.27 (1.69 to 3.13)
	Naik et al^21^	1659	2114	1 to 3 years	OR 1.27 (0.83 to 1.94)	OR 3.30 (0.96 to 11.33)
	Capodanno et al^22^	809	802	1 year	OR 0.74 (0.43 to 1.29) *P*=0.29	OR 2.25 (1.54 to 3.29) *P*<0.001
Randomized controlled trial	Buzman et al^14^	52	53	1 year	1.9% for PCI 7.5% for CABG *P*=0.37	9.7% for PCI 9.4% for CABG *P*=0.97
	SYNTAX substudy^15^	357	348	1 year	4.2% for PCI 4.4% for CABG *P*=0.88	6.5% for PCI 11.8% for CABG *P*=0.02
	Boudriot et al^16^	100	101	1 year	5.0% for PCI 7.98% for CABG	5.9% for PCI 14.0% for CABG
	Park et al^17^	300	300	1 year	4.4% for PCI 4.7% for CABG *P*=0.83	9.0% for PCI 4.2% for CABG *P*=0.02

PCI indicates percutaneous coronary intervention; CABG, coronary artery bypass grafting; TVR, target‐vessel revascularization; TLR, target‐lesion revascularization; SYNTAX, the Synergy between PCI with Taxus and Cardiac Surgery.

**Table 2.** ACC/AHA and ESC Guidelines for Elective PCI for Unprotected Left Main Coronary Artery Disease

Guidelines	COR	LOE
2011 ACC/AHA Guidelines^23^	IIa—For SIHD when both of the following are present:	
	Anatomic conditions associated with a low risk of PCI procedural complications and a high likelihood of good long‐term outcome (eg, a low SYNTAX score of ≤22, ostial or trunk left main stenosis)	B
	Clinical characteristics that predict a significantly increased risk of adverse surgical outcomes (eg, STS‐predicted risk of operative mortality ≥5%)	
	IIb—For SIHD when both of the following are present:	
	Anatomic conditions associated with a low to intermediate risk of PCI procedural complications and an intermediate to high likelihood of good long‐term outcome (eg, low‐intermediate SYNTAX score of <33, bifurcation left main stenosis)	B
	Clinical characteristics that predict an increased risk of adverse surgical outcomes (eg, moderate‐severe COPD, disability from prior stroke, or prior cardiac surgery; STS‐predicted risk of operative mortality >2%)	
	III—For SIHD in patients (vs performing CABG) with unfavorable anatomy for PCI and who are good candidates for CABG	B
2010 ESC Guidelines^24^	IIa—Left main (isolated or 1VD, ostium/shaft)	B
	IIb—Left main (isolated or 1VD, bifurcation)/left main+2VD or 3VD, SYNTAX score ≤32	B
	IIIb—Left main+2VD or 3VD, SYNTAX score ≥33	B

ACC indicates American College of Cardiology; AHA, American Heart Association; ESC, European Society of Cardiology; PCI, percutaneous coronary intervention; COR, class of recommendation; LOE, level of evidence; SIHD, stable ischemic heart disease; SYNTAX, the Synergy between PCI with Taxus and Cardiac Surgery; STS, Society of Thoracic Surgeons; COPD, chronic obstructive pulmonary disease; CABG, coronary artery bypass graft; VD, vessel disease.

## Why Should We Consider FFR in Intermediate LMCA Stenosis?

**Figure 1.**
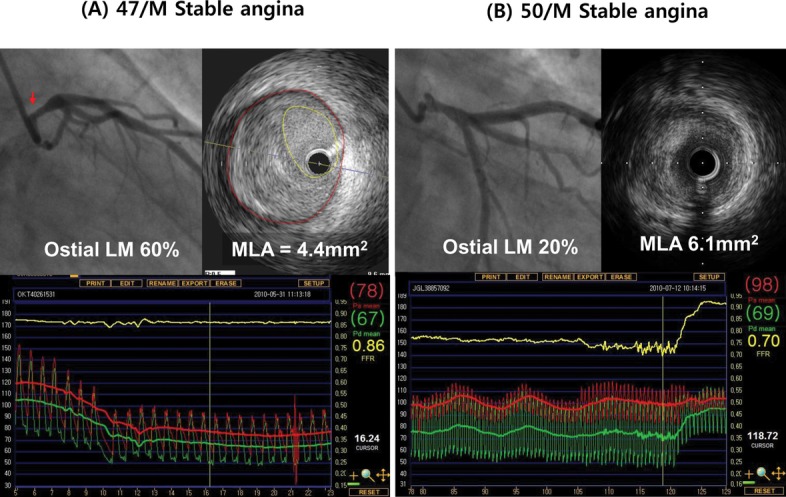
Representative case of visual–functional mismatch in LMCA stenosis. A, Visually estimated percentage diameter stenosis was ≈60%, but FFR was 0.86. B, Visually estimated percentage diameter stenosis was ≈20%, but FFR was 0.70. LM indicates left main; MLA, minimal lumen area; FFR, fractional flow reserve; LMCA, left main coronary artery.

Identification of significant stenosis of LMCA is of critical prognostic importance. Nevertheless, an angiographic stenosis diameter of 50% is still considered a cutoff value for significant LMCA stenosis. Hamilos et al^27^ were the first to demonstrate the considerable discrepancy between coronary angiography and fractional flow reserve (FFR) in the evaluation of intermediate LMCA stenosis. Among the 213 patients in their study, 62 patients (29.1%) showed a “visual functional mismatch” between angiographic significance and functional significance, 13 patients had a diameter stenosis >50% while the FFR was >0.80, and 49 patients had a diameter stenosis <50% while the FFR was <0.80. It is interesting to note that the prevalence of “reverse mismatch,” which refers to angiographically insignificant but functionally significant stenosis, was dominant and as high as 79.0% among the mismatched patients. Figure 1 demonstrates the discrepancy between coronary angiography and FFR.

In addition, noninvasive functional testing such as myocardial perfusion imaging is often noncontributive in the diagnosis of patients with intermediate LMCA stenosis. Perfusion defects are often seen in only 1 vascular territory, or tracer uptake may be reduced in all vascular territories (“balanced ischemia”) giving rise to false‐negative studies, especially when the right coronary artery is significantly diseased.^28^ This is another reason why we should measure FFR for intermediate LMCA stenosis.

Therefore, the decision about whether the treatment of intermediate LMCA stenosis should be performed or deferred should not be determined by coronary angiogram alone, and FFR measurement for intermediate LMCA evaluation should be required, especially in cases of ostial and shaft LMCA disease. FFR measurement could avoid unnecessary LMCA stenting or bypass surgery. Revascularization of a nonsignificant stenosis in the LMCA may lead to early occlusion of the conduits, especially when internal mammary arteries are used.^29^ In addition, when significant LMCA stenosis is highly suspected in a noninvasive functional study in patients with angiographically mild LMCA stenosis, FFR measurement reduced the risk that functionally significant LMCA stenosis remained unrevascularized.

## FFR>0.80 Is a Good Predictor of Favorable Prognosis in Intermediate LMCA Stenosis

An FFR>0.75 to 0.80 has been suggested as a strong predictor of favorable survival and low event rates in patients with intermediate LMCA disease, making it useful for the identification of patients in whom deferral of revascularization is associated with favorable clinical outcomes. In an evaluation of patients with intermediate LMCA stenosis, patients with an FFR≥0.80 treated medically had survival rates comparable to patients with an FFR<0.80 who underwent CABG. Therefore, FFR‐guided decision making for the treatment of intermediate LMCA stenosis is associated with favorable prognosis, and intermediate LMCA disease with an FFR≥0.75 to 0.80 could be safely deferred. Table 3 summarizes key studies that demonstrate FFR‐guided decision making in intermediate LMCA stenosis stenosis.^27,30–33^

**Table 3.** Clinical Outcomes of LMCA Stenosis Managed by FFR‐Guided Decision Making

	Hamilos et al^27^		Bech et al^30^		Courtis et al^31^		Lindstaedt et al^32^		Jasti et al^33^	
Age, y	64±9	68±11	63±9	60±9	61±10	63±10	61±10	64±9	62±11	
Mean follow‐up, months	35±25		29±15		13±10	14±12	29±18	29±14	38	
No. of patients	75	138	30	24	60	82	27	24	14	37
FFR cutoff value	<0.80	≥0.80	<0.75	≥0.75	<0.75*	>0.80	<0.75*	>0.80	<0.75	≥0.75
Treatment	CABG	Medication*	CABG	Medication*	Revascularization (CABG 54, PCI 6)	Medication	CABG	Medication*	Revascularization (CABG 7, PCI 7)	Medication*
Clinical outcomes										
Death, n (%)	7 (9.6)	9 (6.5)	1	0	3 (5)	3 (4)	4 (14.8)	0	0	3 (NC)
Myocardial infarction, n (%)	0	1	1	0	1 (2)	4 (5)	1 (3.7)	0	0	0
Revascularization, n (%)	4 (5.5)	17 (12.3)	2	5	0	9 (11)	1 (3.7)	6 (25)*	0	4

LMCA indicates left main coronary artery; FFR, fractional flow reserve; CABG indicates coronary artery bypass grafting; PCI, percutaneous coronary intervention; NC, noncardiac death.

* Individualized decision was recommended on the basis of additional clinical data if FFR was 0.75 to 0.80.

* Medical treatment or PCI elsewhere in the coronary tree.

* <0.05.

## Conceptual Limitations of FFR in Intermediate LMCA Stenosis and a Practical Approach

**Figure 2.**
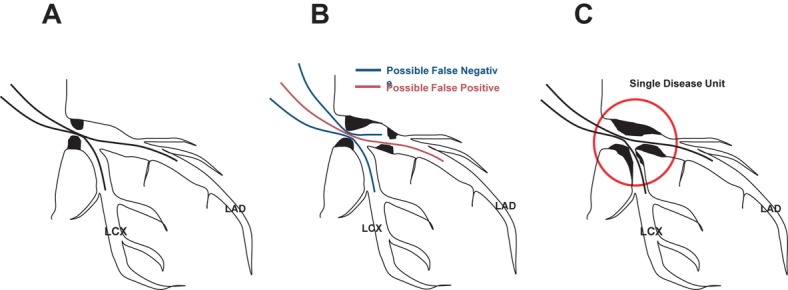
Practical approach for the evaluation of functional significance of left main coronary artery stenosis. LAD indicates left anterior descending artery; LCX, left circumflex artery.

FFR measurement of intermediate ostial and shaft stenosis of LMCA provides accurate information about the functional status of angiographic intermediate stenosis (Figure 2A). However, many physicians have raised questions about the reliability of FFR measurement for the intermediate bifurcation stenosis of LMCA, as they consider the distal LMCA bifurcation as the tandem lesion interposing the large side branch.^34–35^ Therefore, FFR of intermediate LMCA stenosis tends to be under‐ or overestimated because of additional disease in the left anterior descending artery (LAD) and left circumflex artery (LCX) (Figure 2B); thereby, accurate assessment of functional status of intermediate LMCA stenosis itself would not be possible. However, this is only a conceptual limitation. Determining the functional significance and planning for treatment strategy are different issues. Recent IVUS analysis demonstrated the diffuse nature of atherosclerosis involving both the parent (LMCA) segment and both flow dividers (LAD and LCX).^36^ Atherosclerotic plaques extend from the LMCA to the LAD in 90% of patients and LCX in 62% of patients, which suggests distal LMCA bifurcation could not be treated separately and considered as a “single disease unit” (Figure 2C). Therefore, if both FFRs of LAD and LCX side are >0.80, distal LMCA bifurcation is functionally insignificant. If any FFR of LAD and LCX side is ≤0.80, practically, distal LMCA bifurcation PCI as suggested below should be considered, without consideration of the functional significance of intermediate stenosis of the LMCA.

Another reason why the FFR of intermediate LMCA stenosis should be interpreted with caution is that isolated LMCA stenoses are very rare, with most stenoses associated with disease in the LAD and/or LCX, both of which tend to increase FFR measured across the LMCA stenosis. Therefore, in this case, the reassessment for the functional significance of intermediate LMCA stenosis is recommended after the correction of distal coronary artery stenosis.

## Complementary Roles of Intravascular Ultrasound in Functional Evaluation of LMCA Stenosis

Because of the limitations of the conventional coronary angiogram in assessing the severity of LMCA stenosis, there have been several attempts to compare the anatomical parameter assessed by intravascular ultrasound (IVUS) with the corresponding FFR measurement.

Jasti et al^33^ reported that an MLA of 5.9 mm^2^ had the highest sensitivity and specificity (93% and 95%, respectively) for determining a significant LM stenosis, compared with FFR as the gold standard. Recently, clinical application of an MLA criterion for treatment decision making for intermediate LMCA stenosis was tested.^37^ In the LITRO study, a total of 354 patients with intermediate LMCA stenoses were enrolled. In patients with an MLA<6 mm^2^, revascularization were performed, and in patients with an MLA≥6 mm^2^, revascularization was deferred. In a 2‐year follow‐up period, there were no statistical between‐group differences regarding the incidence of death (2.3% versus 4.5%, respectively; *P*=0.5) and any event (12.7% versus 19.4%, respectively; *P*=0.3). Therefore, they suggested an MLA ≥6 mm^2^ was a safe value for deferring revascularization of the LMCA.

**Figure 3.**
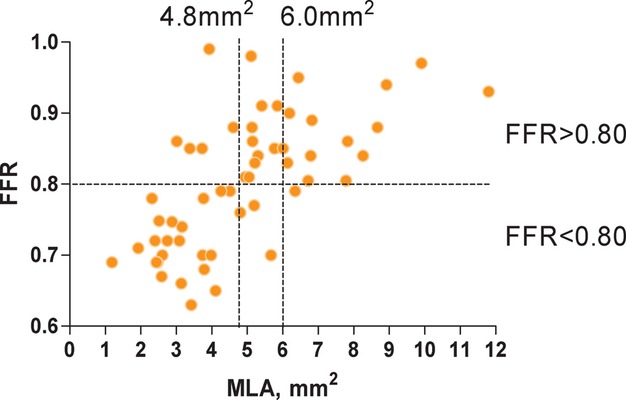
Correlation between minimal lumen area and fractional flow reserve in intermediate left main coronary artery stenosis. FFR indicates fractional flow reserve. With permission from Kang et al.^38^

We recently addressed these issues in 55 patients with isolated intermediate LMCA stenosis who underwent preinterventional IVUS and FFR measurements to determine the IVUS MLA criterion corresponding to an FFR<0.80.^38^ We found that the IVUS MLA value within the LMCA that best predicted FFR<0.80 was <4.8 mm^2^ (89% sensitivity, 83% specificity, 86% accuracy; AUC 0.90, 95% CI 0.788 to 0.964, *P*<0.001). It is interesting to note that that the positive predictive value of IVUS‐measured MLA<4.8 mm^2^ is acceptably high at 82%, in contrast with non‐LMCA stenosis (Figure 3).^38^ This might be explained by the simplicity of morphological characteristics of pure LM lesions, uniformly large vessel size, short lesion length, and lack of side branch and other anatomical factors that could potentially affect FFR. Therefore, in the evaluation of intermediate LMCA stenosis, anatomical parameter provided by IVUS appeared to be correlated well with functional significance of LMCA stenosis.

## How to Perform Unprotected LMCA Stenting

From a technical perspective, it would be easy to perform a single‐stent procedure for ostial and shaft LM disease; published long‐term clinical outcomes are excellent.^39–41^ The current PCI guideline was updated as mentioned before.^23–24^ For LMCA bifurcation disease, unresolved technical issues remain. The single‐stent technique clearly shows more favorable long‐term clinical outcomes compared with the 2‐stent technique, even in bifurcation LM disease.^42–45^ Therefore, in real practice, the single‐stent crossover technique has been used more frequently, in as many as ≈60% of all LMCA bifurcation treatments.^42^ Selection of a single‐ or 2‐stent technique should be based on disease involvement of the LCX ostium, because side‐branch compromise after stent crossover is frequent in the setting of significant ostial disease of the side branch (Table 4). Thus, to determine the choice of a single‐ or 2‐stent strategy, IVUS provides accurate information for both main‐ and side‐branch disease status and vascular remodeling in LMCA bifurcation lesions. In addition, if possible, direct imaging from the LCX is necessary for accurate assessment of the side branch, including its ostium, because IVUS evaluation of a side‐branch ostium from the main vessel is only moderately reliable.^46^

**Table 4.** Favorable or Unfavorable Anatomical Features for Single‐Stent Crossover Stenting in Treatment of Unprotected Left Main Coronary Artery Stenosis^54^

	Anatomical Features
Favorable	Insignificant stenosis at the ostial LCX with Medina classification 1,1,0 or 1,0,0
	Diminutive LCX with <2.5 mm in diameter; right dominant coronary system
	Wide angle with LAD
	No concomitant disease in LCX
	Focal disease in LCX
Unfavorable	Insignificant stenosis at the ostial LCX with Medina classification 1,1,1; 1,0,1; or 0,1,1
	Large size of LCX with ≥2.5 mm in diameter; left dominant coronary system
	Narrow angle with LAD
	Concomitant disease in LCX
	Diffuse disease in LCX

LCX indicates left circumex artery; LAD, left anterior descending artery.

Table is adapted with permission from Moussa et al.^54^

**Figure 4.**
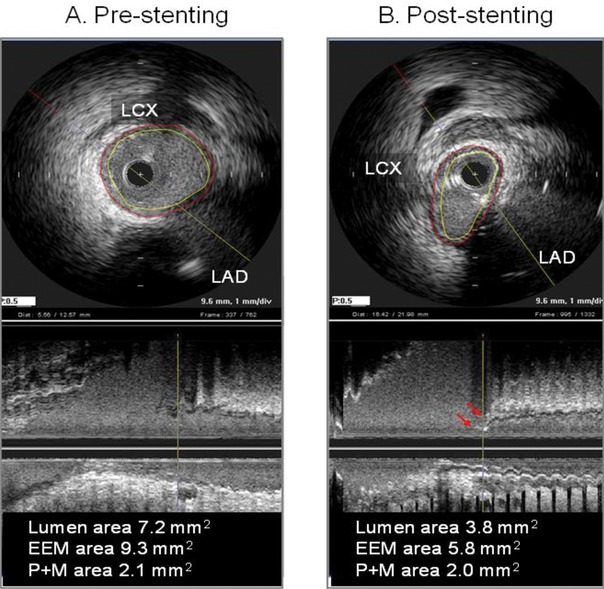
Geometric changes in left main coronary artery bifurcation after main‐branch stenting. Longitudinal image reconstruction demonstrated carina shift into the LCX poststenting (arrow). EEM indicates external elastic membrane; P+M, plaque area plus media area; LAD, left anterior descending artery; LCX, left circumflex artery. Adapted with permission from Kang et al.^47^

After main‐stent crossover from the proximal left anterior descending artery (LAD) to LM, geometric changes in the LCX ostium were related mainly to carina shift, reduction of MLA, and increased eccentricity of the external elastic membrane and carina angle between the LAD and the LCX (Figure 4).^47^ However, an important issue is being unable to predict the functional significance of the stenosis with only the degree of jailed LCX ostium, no matter how big or small. Therefore, in cases in which the LCX ostium is significantly compromised (>50%) after simple crossover stent implantation from LM to LAD, we should consider FFR measurement first before further treatment of the LCX.

## IVUS Minimal Stent Area Criteria Optimizing the Clinical Outcomes

Optimal stent expansion was considered one of the most important factors in preventing restenosis or adverse clinical outcomes.^48–49^ However, there are no data suggesting the optimal minimal stent area (MSA) cutoff for prediction of restenosis and long‐term clinical outcome after DES implantation for LMCA stenosis.

**Figure 5.**
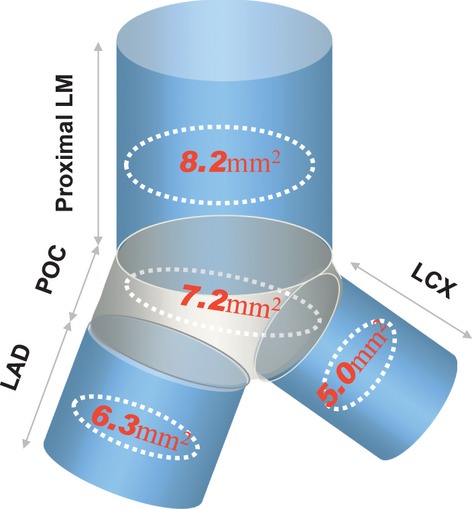
Cutoff values of minimal stent area for the prediction of angiographic in‐stent restenosis on a segmental basis. LM indicates left main; POC, polygon of confluence; LAD, left anterior descending artery; LCX, left circumflex artery. Adapted with permission from Kang et al.^50^

Recently, we studied the optimal IVUS‐MSA criteria for prevention of in‐stent restenosis (ISR) in 403 patients undergoing sirolimus‐eluting stent implantation for LMCA disease.^50^ We classified the LMCA into 4 segments: the LCX ostium, LAD ostium, polygon of confluence (POC), and LMCA above the POC. The best IVUS‐MSA criteria that predicted angiographic ISR on a segmental basis were 5.0 mm^2^ for the LCX ostium, 6.3 mm^2^ for the LAD ostium, 7.2 mm^2^ for the POC, and 8.2 mm^2^ for the proximal LMCA above the POC (Figure 5).^50^ Using these criteria, 133 patients (33.8%) experienced underexpansion ≥1 of the prespecified segments. In addition, underexpansion was more frequent in the 2‐stent group than in the single‐stent group (54% versus 27%, respectively, *P*=0.001). In the 2‐stent group, the LCX ostium was the most common site of underexpansion (37%), which may explain the greater risk of ISR when LMCA bifurcation lesions are treated with a 2‐stent strategy. Overall, angiographic ISR was more frequent in lesions with underexpansion than in lesions without underexpansion (24.1% versus 5.4%, respectively; *P*=0.001). Even in the 2‐stent group, lesions with complete expansion at all sites showed only 6% of the ISR rate, which was similar to that of the single‐stent group (6.3%) or in nonbifurcation LMCA lesions (4.5%). Furthermore, a smaller IVUS‐MSA predicted angiographic ISR 9 months after DES implantation for treatment of LMCA disease, and poststenting underexpansion was an independent predictor of 2‐year adverse clinical outcomes, especially repeat revascularization.

## Impact of IVUS Guidance for LMCA Stenting

Although IVUS guidance has been useful in stenting unprotected LMCA stenoses, its impact on long‐term mortality is still unclear. In 201 matched pairs from the MAIN‐COMPARE registry, there was a tendency of lower risk for 3‐year mortality with IVUS guidance compared with angiography guidance (6.0% versus 13.6%, respectively; log‐rank *P*=0.063; hazard ratio 0.54).^26^ In particular, for 145 matched pairs of patients receiving DES, the 3‐year mortality was significantly lower for IVUS guidance compared with angiography guidance (4.7% versus 16.0%, respectively; log‐rank *P*=0.048; hazard ratio 0.39). It is interesting to note that the mortality rate started to diverge beyond 1 year after the procedure. In contrast, the use of IVUS did not reduce the risk of mortality in 47 matched pairs of patients receiving a bare‐metal stent (8.6% versus 10.8%, respectively; log‐rank *P*=0.35; hazard ratio 0.59). Therefore, despite inherent limitations of nonrandomized registry design, this study indicated that IVUS guidance may play a role in reducing very late stent thrombosis and subsequent long‐term mortality.

IVUS guidance has provided more information on negative remodeling, reference vessel size, and morphologic complexity of ostial or bifurcation lesions in preintervention evaluation, stent underexpansion, incomplete lesion coverage, small stent area, large residual plaque, and incomplete stent apposition in postinterventional evaluation.^51–53^ For LMCA lesions, in particular, the use of IVUS is helpful in determining treatment strategy and in optimizing the stent procedure. Therefore, we strongly recommend mandatory use of IVUS in PCI for unprotected LMCA.

## Conclusions

**Figure 6.**
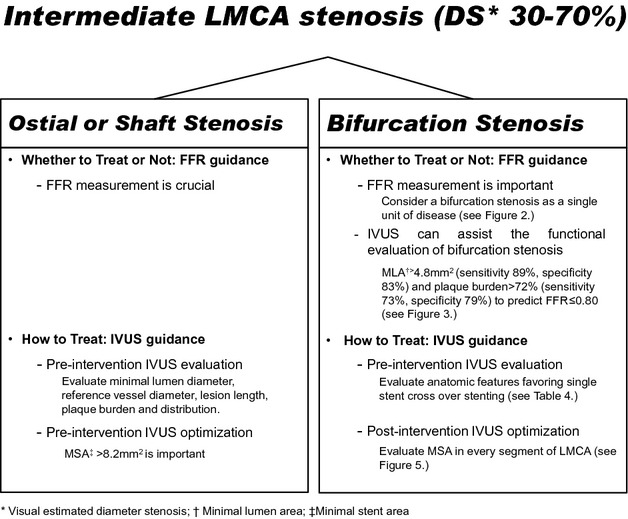
Integrated use of FFR and IVUS in left main stenting. LMCA indicates left main coronary artery; FFR, fractional flow reserve; IVUS, intravascular ultrasound; MLA, minimal lumen area; MSA, minimal stent area.

FFR‐guided PCI can help to select appropriate patients and lesions for treatment, avoid unnecessary procedures, reduce medical costs, and improve clinical outcomes. Furthermore, IVUS can be used to secure the PCI procedure by preinterventional lesion assessment and postinterventional stent optimization. We propose the concept of the integrated use of FFR and IVUS in LMCA stenting (Figure 6). Despite several limitations of this approach including cost, procedural time, and availability of trained personnel, FFR‐guided complex PCI, which is supported by IVUS, can give us better insights into LMCA disease and may improve the clinical outcomes of patients who undergo LMCA stenting.

## Disclosure

None.

## References

[1] Chaitman BR Fisher LD Bourassa MG Davis K Rogers WJ Maynard C Tyras DH Berger RL Judkins MP Ringqvist I Mock MB Killip T Effect of coronary bypass surgery on survival patterns in subsets of patients with left main coronary artery disease. Report of the Collaborative Study in Coronary Artery Surgery (CASS). Am J Cardiol. 1981; 48:765-777 7025604 10.1016/0002-9149(81)90156-9

[2] Takaro T Peduzzi P Detre KM Hultgren HN Murphy ML van der Bel‐Kahn J Thomsen J Meadows WR Survival in subgroups of patients with left main coronary artery disease. Veterans Administration Cooperative Study of Surgery for Coronary Arterial Occlusive Disease. Circulation. 1982; 66:14-22 6979435 10.1161/01.cir.66.1.14

[3] Park SJ Park DW Percutaneous coronary intervention with stent implantation versus coronary artery bypass surgery for treatment of left main coronary artery disease: is it time to change guidelines? Circ Cardiovasc Interv. 2009; 2:59-68 20031694 10.1161/CIRCINTERVENTIONS.108.831701

[4] Park DW Seung KB Kim YH Lee JY Kim WJ Kang SJ Lee SW Lee CW Park SW Yun SC Gwon HC Jeong MH Jang YS Kim HS Kim PJ Seong IW Park HS Ahn T Chae IH Tahk SJ Chung WS Park SJ Long‐term safety and efficacy of stenting versus coronary artery bypass grafting for unprotected left main coronary artery disease: 5‐year results from the MAIN‐COMPARE (Revascularization for Unprotected Left Main Coronary Artery Stenosis: comparison of Percutaneous Coronary Angioplasty Versus Surgical Revascularization) registry. J Am Coll Cardiol. 2010; 56:117-124 20451344 10.1016/j.jacc.2010.04.004

[5] Lee MS Kapoor N Jamal F Czer L Aragon J Forrester J Kar S Dohad S Kass R Eigler N Trento A Shah PK Makkar RR Comparison of coronary artery bypass surgery with percutaneous coronary intervention with drug‐eluting stents for unprotected left main coronary artery disease. J Am Coll Cardiol. 2006; 47:864-870 16487857 10.1016/j.jacc.2005.09.072

[6] Chieffo A Stankovic G Bonizzoni E Tsagalou E Iakovou I Montorfano M Airoldi F Michev I Sangiorgi MG Carlino M Vitrella G Colombo A Early and mid‐term results of drug‐eluting stent implantation in unprotected left main. Circulation. 2005; 111:791-795 15699254 10.1161/01.CIR.0000155256.88940.F8

[7] Palmerini T Marzocchi A Marrozzini C Ortolani P Saia F Savini C Bacchi‐Reggiani L Gianstefani S Virzi S Manara F Kiros Weldeab M Marinelli G Di Bartolomeo R Branzi A Comparison between coronary angioplasty and coronary artery bypass surgery for the treatment of unprotected left main coronary artery stenosis (the Bologna Registry). Am J Cardiol. 2006; 98:54-59 16784920 10.1016/j.amjcard.2006.01.070

[8] Sanmartin M Baz JA Claro R Asorey V Duran D Pradas G Iniguez A Comparison of drug‐eluting stents versus surgery for unprotected left main coronary artery disease. Am J Cardiol. 2007; 100:970-973 17826380 10.1016/j.amjcard.2007.04.037

[9] Makikallio TH Niemela M Kervinen K Jokinen V Laukkanen J Ylitalo I Tulppo MP Juvonen J Huikuri HV Coronary angioplasty in drug eluting stent era for the treatment of unprotected left main stenosis compared to coronary artery bypass grafting. Ann Med. 2008; 40:437-443 18608116 10.1080/07853890701879790

[10] Cheng CI Lee FY Chang JP Hsueh SK Hsieh YK Fang CY Chen SM Yang CH Yip HK Chen MC Fu M Wu CJ Long‐term outcomes of intervention for unprotected left main coronary artery stenosis: coronary stenting vs coronary artery bypass grafting. Circ J. 2009; 73:705-712 19261993 10.1253/circj.cj-08-0804

[11] Wu X Chen Y Liu H Teirstein PS Kirtane AJ Ge C Song X Chen X Gu C Huang F Lv S Comparison of long‐term (4‐year) outcomes of patients with unprotected left main coronary artery narrowing treated with drug‐eluting stents versus coronary‐artery bypass grafting. Am J Cardiol. 2010; 105:1728-1734 20538122 10.1016/j.amjcard.2010.01.353

[12] Park DW Kim YH Yun SC Lee JY Kim WJ Kang SJ Lee SW Lee CW Kim JJ Choo SJ Chung CH Lee JW Park SW Park SJ Long‐term outcomes after stenting versus coronary artery bypass grafting for unprotected left main coronary artery disease: 10‐year results of bare‐metal stents and 5‐year results of drug‐eluting stents from the ASAN‐MAIN (ASAN Medical Center‐Left MAIN Revascularization) Registry. J Am Coll Cardiol. 2010; 56:1366-1375 20946993 10.1016/j.jacc.2010.03.097

[13] Caggegi A Capodanno D Capranzano P Chisari A Ministeri M Mangiameli A Ronsivalle G Ricca G Barrano G Monaco S Di Salvo ME Tamburino C Comparison of one‐year outcomes of percutaneous coronary intervention versus coronary artery bypass grafting in patients with unprotected left main coronary artery disease and acute coronary syndromes (from the CUSTOMIZE Registry). Am J Cardiol. 2011; 108:355-359 21545992 10.1016/j.amjcard.2011.03.050

[14] Buszman PE Kiesz SR Bochenek A Peszek‐Przybyla E Szkrobka I Debinski M Bialkowska B Dudek D Gruszka A Zurakowski A Milewski K Wilczynski M Rzeszutko L Buszman P Szymszal J Martin JL Tendera M Acute and late outcomes of unprotected left main stenting in comparison with surgical revascularization. J Am Coll Cardiol. 2008; 51:538-545 18237682 10.1016/j.jacc.2007.09.054

[15] Morice MC Serruys PW Kappetein AP Feldman TE Stahle E Colombo A Mack MJ Holmes DR Torracca L van Es GA Leadley K Dawkins KD Mohr F Outcomes in patients with de novo left main disease treated with either percutaneous coronary intervention using paclitaxel‐eluting stents or coronary artery bypass graft treatment in the Synergy Between Percutaneous Coronary Intervention with TAXUS and Cardiac Surgery (SYNTAX) trial. Circulation. 2010; 121:2645-2653 20530001 10.1161/CIRCULATIONAHA.109.899211

[16] Boudriot E Thiele H Walther T Liebetrau C Boeckstegers P Pohl T Reichart B Mudra H Beier F Gansera B Neumann FJ Gick M Zietak T Desch S Schuler G Mohr FW Randomized comparison of percutaneous coronary intervention with sirolimus‐eluting stents versus coronary artery bypass grafting in unprotected left main stem stenosis. J Am Coll Cardiol. 2011; 57:538-545 21272743 10.1016/j.jacc.2010.09.038

[17] Park JS Choi YW Shin JS Yang HM Lim HS Choi BJ Choi SY Yoon MH Hwang GS Tahk SJ Shin JH Validation of three‐dimensional echocardiography for quantification of aortic root geometry: comparison with multi‐detector computed tomography. J Cardiovasc Ultrasound. 2011; 19:128-133 22073322 10.4250/jcu.2011.19.3.128 PMC3209591

[18] Seung KB Park DW Kim YH Lee SW Lee CW Hong MK Park SW Yun SC Gwon HC Jeong MH Jang Y Kim HS Kim PJ Seong IW Park HS Ahn T Chae IH Tahk SJ Chung WS Park SJ Stents versus coronary‐artery bypass grafting for left main coronary artery disease. N Engl J Med. 2008; 358:1781-1792 18378517 10.1056/NEJMoa0801441

[19] Takagi H Kawai N Umemoto T Stenting versus coronary artery bypass grafting for unprotected left main coronary artery disease: a meta‐analysis of comparative studies. J Thorac Cardiovasc Surg. 2009; 137:e54-e57 19154888 10.1016/j.jtcvs.2008.06.006

[20] Lee MS Yang T Dhoot J Liao H Meta‐analysis of clinical studies comparing coronary artery bypass grafting with percutaneous coronary intervention and drug‐eluting stents in patients with unprotected left main coronary artery narrowings. Am J Cardiol. 2010; 105:1070-1075 20381655 10.1016/j.amjcard.2009.12.007

[21] Naik H White AJ Chakravarty T Forrester J Fontana G Kar S Shah PK Weiss RE Makkar R A meta‐analysis of 3,773 patients treated with percutaneous coronary intervention or surgery for unprotected left main coronary artery stenosis. JACC Cardiovasc Interv. 2009; 2:739-747 19695542 10.1016/j.jcin.2009.05.020

[22] Capodanno D Stone GW Morice MC Bass TA Tamburino C Percutaneous coronary intervention versus coronary artery bypass graft surgery in left main coronary artery disease: a meta‐analysis of randomized clinical data. J Am Coll Cardiol. 2011; 58:1426-1432 21939824 10.1016/j.jacc.2011.07.005

[23] Levine GN Bates ER Blankenship JC Bailey SR Bittl JA Cercek B Chambers CE Ellis SG Guyton RA Hollenberg SM Khot UN Lange RA Mauri L Mehran R Moussa ID Mukherjee D Nallamothu BK Ting HH 2011 ACCF/AHA/SCAI Guideline for Percutaneous Coronary Intervention: a report of the American College of Cardiology Foundation/American Heart Association Task Force on Practice Guidelines and the Society for Cardiovascular Angiography and Interventions. Circulation. 2011; 124:e574-e651 22064601 10.1161/CIR.0b013e31823ba622

[24] Wijns W Kolh P Danchin N Di Mario C Falk V Folliguet T Garg S Huber K James S Knuuti J Lopez‐Sendon J Marco J Menicanti L Ostojic M Piepoli MF Pirlet C Pomar JL Reifart N Ribichini FL Schalij MJ Sergeant P Serruys PW Silber S Sousa Uva M Taggart D Vahanian A Auricchio A Bax J Ceconi C Dean V Filippatos G Funck‐Brentano C Hobbs R Kearney P McDonagh T Popescu BA Reiner Z Sechtem U Sirnes PA Tendera M Vardas PE Widimsky P Alfieri O Dunning J Elia S Kappetein P Lockowandt U Sarris G Vouhe P von Segesser L Agewall S Aladashvili A Alexopoulos D Antunes MJ Atalar E Brutel de la Riviere A Doganov A Eha J Fajadet J Ferreira R Garot J Halcox J Hasin Y Janssens S Kervinen K Laufer G Legrand V Nashef SA Neumann FJ Niemela K Nihoyannopoulos P Noc M Piek JJ Pirk J Rozenman Y Sabate M Starc R Thielmann M Wheatley DJ Windecker S Zembala M Guidelines on myocardial revascularization: The Task Force on Myocardial Revascularization of the European Society of Cardiology (ESC) and the European Association for Cardio‐Thoracic Surgery (EACTS). Eur Heart J. 2010; 31:2501-2555 20802248 10.1093/eurheartj/ehq277

[25] Riley RF Don CW Powell W Maynard C Dean LS Trends in coronary revascularization in the United States from 2001 to 2009: recent declines in percutaneous coronary intervention volumes. Circ Cardiovasc Qual Outcomes. 2011; 4:193-197 21304092 10.1161/CIRCOUTCOMES.110.958744 PMC3072819

[26] Park SJ Kim YH Park DW Lee SW Kim WJ Suh J Yun SC Lee CW Hong MK Lee JH Park SW Impact of intravascular ultrasound guidance on long‐term mortality in stenting for unprotected left main coronary artery stenosis. Circ Cardiovasc Interv. 2009; 2:167-177 20031713 10.1161/CIRCINTERVENTIONS.108.799494

[27] Hamilos M Muller O Cuisset T Ntalianis A Chlouverakis G Sarno G Nelis O Bartunek J Vanderheyden M Wyffels E Barbato E Heyndrickx GR Wijns W De Bruyne B Long‐term clinical outcome after fractional flow reserve‐guided treatment in patients with angiographically equivocal left main coronary artery stenosis. Circulation. 2009; 120:1505-1512 19786633 10.1161/CIRCULATIONAHA.109.850073

[28] Ragosta M Bishop AH Lipson LC Watson DD Gimple LW Sarembock IJ Powers ER Comparison between angiography and fractional flow reserve versus single‐photon emission computed tomographic myocardial perfusion imaging for determining lesion significance in patients with multivessel coronary disease. Am J Cardiol. 2007; 99:896-902 17398179 10.1016/j.amjcard.2006.11.035

[29] Botman CJ Schonberger J Koolen S Penn O Botman H Dib N Eeckhout E Pijls N Does stenosis severity of native vessels influence bypass graft patency? A prospective fractional flow reserve‐guided study. Ann Thorac Surg. 2007; 83:2093-2097 17532405 10.1016/j.athoracsur.2007.01.027

[30] Bech GJ Droste H Pijls NH De Bruyne B Bonnier JJ Michels HR Peels KH Koolen JJ Value of fractional flow reserve in making decisions about bypass surgery for equivocal left main coronary artery disease. Heart. 2001; 86:547-552 11602550 10.1136/heart.86.5.547 PMC1729979

[31] Courtis J Rodes‐Cabau J Larose E Potvin JM Dery JP Larochelliere RD Cote M Cousterousse O Nguyen CM Proulx G Rinfret S Bertrand OF Usefulness of coronary fractional flow reserve measurements in guiding clinical decisions in intermediate or equivocal left main coronary stenoses. Am J Cardiol. 2009; 103:943-949 19327420 10.1016/j.amjcard.2008.11.054

[32] Lindstaedt M Yazar A Germing A Fritz MK Holland‐Letz T Mugge A Bojara W Clinical outcome in patients with intermediate or equivocal left main coronary artery disease after deferral of surgical revascularization on the basis of fractional flow reserve measurements. Am Heart J. 2006; 152:156.e151-156.e159 16824848 10.1016/j.ahj.2006.03.026

[33] Jasti V Ivan E Yalamanchili V Wongpraparut N Leesar MA Correlations between fractional flow reserve and intravascular ultrasound in patients with an ambiguous left main coronary artery stenosis. Circulation. 2004; 110:2831-2836 15492302 10.1161/01.CIR.0000146338.62813.E7

[34] Pijls NH De Bruyne B Bech GJ Liistro F Heyndrickx GR Bonnier HJ Koolen JJ Coronary pressure measurement to assess the hemodynamic significance of serial stenoses within one coronary artery: validation in humans. Circulation. 2000; 102:2371-2377 11067791 10.1161/01.cir.102.19.2371

[35] De Bruyne B Pijls NH Heyndrickx GR Hodeige D Kirkeeide R Gould KL Pressure‐derived fractional flow reserve to assess serial epicardial stenoses: theoretical basis and animal validation. Circulation. 2000; 101:1840-1847 10769286 10.1161/01.cir.101.15.1840

[36] Oviedo C Maehara A Mintz GS Araki H Choi SY Tsujita K Kubo T Doi H Templin B Lansky AJ Dangas G Leon MB Mehran R Tahk SJ Stone GW Ochiai M Moses JW Intravascular ultrasound classification of plaque distribution in left main coronary artery bifurcations: where is the plaque really located? Circ Cardiovasc Interv. 2010; 3:105-112 20197513 10.1161/CIRCINTERVENTIONS.109.906016

[37] de la Torre Hernandez JM Hernandez Hernandez F Alfonso F Rumoroso JR Lopez‐Palop R Sadaba M Carrillo P Rondan J Lozano I Ruiz Nodar JM Baz JA Fernandez Nofrerias E Pajin F Garcia Camarero T Gutierrez H Group LS Prospective application of pre‐defined intravascular ultrasound criteria for assessment of intermediate left main coronary artery lesions results from the multicenter LITRO study. J Am Coll Cardiol. 2011; 58:351-358 21757111 10.1016/j.jacc.2011.02.064

[38] Kang SJ Lee JY Ahn JM Song HG Kim WJ Park DW Yun SC Lee SW Kim YH Mintz GS Lee CW Park SW Park SJ Intravascular ultrasound‐derived predictors for fractional flow reserve in intermediate left main disease. JACC Cardiovasc Interv. 2011; 4:1168-1174 22115656 10.1016/j.jcin.2011.08.009

[39] Chieffo A Park SJ Valgimigli M Kim YH Daemen J Sheiban I Truffa A Montorfano M Airoldi F Sangiorgi G Carlino M Michev I Lee CW Hong MK Park SW Moretti C Bonizzoni E Rogacka R Serruys PW Colombo A Favorable long‐term outcome after drug‐eluting stent implantation in nonbifurcation lesions that involve unprotected left main coronary artery: a multicenter registry. Circulation. 2007; 116:158-162 17576862 10.1161/CIRCULATIONAHA.107.692178

[40] Park DW Hong MK Suh IW Hwang ES Lee SW Jeong YH Kim YH Lee CW Kim JJ Park SW Park SJ Results and predictors of angiographic restenosis and long‐term adverse cardiac events after drug‐eluting stent implantation for aorto‐ostial coronary artery disease. Am J Cardiol. 2007; 99:760-765 17350360 10.1016/j.amjcard.2006.10.028

[41] Lee SW Kim SH Kim SO Han S Kim YH Park DW Kang SJ Lee CW Park SW Park SJ Comparative long‐term efficacy and safety of drug‐eluting stent versus coronary artery bypass grafting in ostial left main coronary artery disease: analysis of the MAIN‐COMPARE Registry. Catheter Cardiovasc Interv. 2012; 80:206-212 22234884 10.1002/ccd.23369

[42] Kim WJ Kim YH Park DW Yun SC Lee JY Kang SJ Lee SW Lee CW Park SW Park SJ Comparison of single‐ versus two‐stent techniques in treatment of unprotected left main coronary bifurcation disease. Catheter Cardiovasc Interv. 2011; 77:775-782 21520380 10.1002/ccd.22915

[43] Kim YH Park SW Hong MK Park DW Park KM Lee BK Song JM Han KH Lee CW Kang DH Song JK Kim JJ Park SJ Comparison of simple and complex stenting techniques in the treatment of unprotected left main coronary artery bifurcation stenosis. Am J Cardiol. 2006; 97:1597-1601 16728221 10.1016/j.amjcard.2005.12.051

[44] Valgimigli M Malagutti P Rodriguez Granillo GA Tsuchida K Garcia‐Garcia HM van Mieghem CA Van der Giessen WJ De Feyter P de Jaegere P Van Domburg RT Serruys PW Single‐vessel versus bifurcation stenting for the treatment of distal left main coronary artery disease in the drug‐eluting stenting era. Clinical and angiographic insights into the Rapamycin‐Eluting Stent Evaluated at Rotterdam Cardiology Hospital (RESEARCH) and Taxus‐Stent Evaluated at Rotterdam Cardiology Hospital (T‐SEARCH) registries. Am Heart J. 2006; 152:896-902 17070153 10.1016/j.ahj.2006.03.029

[45] Palmerini T Marzocchi A Tamburino C Sheiban I Margheri M Vecchi G Sangiorgi G Santarelli A Bartorelli A Briguori C Vignali L Di Pede F Ramondo A Inglese L De Carlo M Falsini G Benassi A Palmieri C Filippone V Sangiorgi D Barlocco F De Servi S Impact of bifurcation technique on 2‐year clinical outcomes in 773 patients with distal unprotected left main coronary artery stenosis treated with drug‐eluting stents. Circ Cardiovasc Interv. 2008; 1:185-192 20031677 10.1161/CIRCINTERVENTIONS.108.800631

[46] Kang SJ Mintz GS Oh JH Park DW Lee SW Kim YH Lee CW Park SW Park SJ Intravascular ultrasound assessment of distal left main bifurcation disease: the importance of the polygon of confluence of the left main, left anterior descending, and left circumflex arteries. Catheter Cardiovasc Interv. 201110.1002/ccd.23263 10.1002/ccd.23263 21805589

[47] Kang SJ Mintz GS Kim WJ Lee JY Oh JH Park DW Lee SW Kim YH Lee CW Park SW Park SJ Changes in left main bifurcation geometry after a single‐stent crossover technique: an intravascular ultrasound study using direct imaging of both the left anterior descending and the left circumflex coronary arteries before and after intervention. Circ Cardiovasc Interv. 2011; 4:355-361 21712525 10.1161/CIRCINTERVENTIONS.110.961045

[48] Hong MK Mintz GS Lee CW Park DW Choi BR Park KH Kim YH Cheong SS Song JK Kim JJ Park SW Park SJ Intravascular ultrasound predictors of angiographic restenosis after sirolimus‐eluting stent implantation. Eur Heart J. 2006; 27:1305-1310 16682378 10.1093/eurheartj/ehi882

[49] Sonoda S Morino Y Ako J Terashima M Hassan AH Bonneau HN Leon MB Moses JW Yock PG Honda Y Kuntz RE Fitzgerald PJ Impact of final stent dimensions on long‐term results following sirolimus‐eluting stent implantation: serial intravascular ultrasound analysis from the sirius trial. J Am Coll Cardiol. 2004; 43:1959-1963 15172398 10.1016/j.jacc.2004.01.044

[50] Kang SJ Ahn JM Song H Kim WJ Lee JY Park DW Yun SC Lee SW Kim YH Lee CW Mintz GS Park SW Park SJ Comprehensive intravascular ultrasound assessment of stent area and its impact on restenosis and adverse cardiac events in 403 patients with unprotected left main disease. Circ Cardiovasc Interv. 2011; 4:562-569 22045969 10.1161/CIRCINTERVENTIONS.111.964643

[51] Fujii K Carlier SG Mintz GS Yang Y‐M Moussa I Weisz G Dangas G Mehran R Lansky AJ Kreps EM Collins M Stone GW Moses JW Leon MB Stent underexpansion and residual reference segment stenosis are related to stent thrombosis after sirolimus‐eluting stent implantation: An intravascular ultrasound study. J Am Coll Cardiol. 2005; 45:995-998 15808753 10.1016/j.jacc.2004.12.066

[52] Cook S Wenaweser P Togni M Billinger M Morger C Seiler C Vogel R Hess O Meier B Windecker S Incomplete stent apposition and very late stent thrombosis after drug‐eluting stent implantation. Circulation. 2007; 115:2426-2434 17485593 10.1161/CIRCULATIONAHA.106.658237

[53] Okabe T Mintz GS Buch AN Roy P Hong YJ Smith KA Torguson R Gevorkian N Xue Z Satler LF Kent KM Pichard AD Weissman NJ Waksman R Intravascular ultrasound parameters associated with stent thrombosis after drug‐eluting stent deployment. Am J Cardiol. 2007; 100:615-620 17697816 10.1016/j.amjcard.2007.03.072

[54] Moussa ID Colombo A Tips and Tricks in Interventional Therapy of Coronary Bifurcation Lesions. 2010 1st edition London, UK Informa healthcare 135

